# Liposome-Loaded Mesenchymal Stem Cells Enhance Tumor Accumulation and Anti-Tumor Efficacy of Doxorubicin in Mouse Tumor Models of Melanoma

**DOI:** 10.3390/pharmaceutics17080947

**Published:** 2025-07-22

**Authors:** Yusuke Kono, Himi Kanbara, Saki Danjo, Aiga Yoshikawa, Yoshihiro Iwayama, Ken-ichi Ogawara

**Affiliations:** Laboratory of Pharmaceutics, Kobe Pharmaceutical University, 4-19-1 Motoyamakita-machi, Higashinada-ku, Kobe 658-8558, Japan; hi202304@st.kobepharma-u.ac.jp (H.K.); jt200077@st.kobepharma-u.ac.jp (S.D.); gx212822@st.kobepharma-u.ac.jp (A.Y.); nt201341@st.kobepharma-u.ac.jp (Y.I.); ohgawa-k@kobepharma-u.ac.jp (K.-i.O.)

**Keywords:** mesenchymal stem cell, liposome, drug delivery, tumor targeting, doxorubicin

## Abstract

**Background**: Mesenchymal stem cells (MSCs) possess an intrinsic tumor-tropic ability, and therefore, MSCs may potentially be used as biomimetic carriers for active drug delivery systems targeting tumors. We previously developed a method to efficiently load liposomes onto the surface of MSCs via electrostatic interactions. The prepared liposome-loaded MSCs (Lip-MSCs) spontaneously accumulated in solid melanoma tumors with low vascular permeability while stably carrying liposomes. **Methods**: To explore Lip-MSC applications in cancer chemotherapy, doxorubicin (DOX)-encapsulated liposomes (DOX-Lip) were prepared and loaded onto MSCs. The cell viability, DOX-releasing properties, tumor-homing capacity, and anti-tumor efficacy of DOX-Lip-MSCs were analyzed. **Results**: Small liposomes (100 nm) retained DOX, whereas significant leakage of DOX was observed from 600 nm-sized liposomes. Based on this result, we used 100 nm DOX-Lip for the preparation of DOX-Lip-MSCs. Compared with MSCs loaded with DOX by incubation with DOX solution, DOX-Lip-MSCs could load a larger amount of DOX with minimal cytotoxicity. DOX-Lip-MSCs also showed sustained DOX release. DOX-Lip-MSCs efficiently migrated toward the conditioned medium of B16/BL6 melanoma cells in vitro and accumulated in B16/BL6 tumors in vivo, leading to a significant inhibitory effect on tumor growth. **Conclusions**: Lip-MSCs can serve as an efficient carrier to deliver anti-cancer drugs into solid tumors.

## 1. Introduction

Nano-formulations, such as liposomes, lipid nanoparticles and emulsions, are clinically used as carriers in drug delivery systems for cancer therapy [1,2]. Nanoparticles smaller than 100 nm passively accumulate in tumor tissue based on the enhanced permeability and retention (EPR) effect. However, the tumor tissues from patients with some intractable cancers, including melanoma, lung, and pancreatic cancer, have abundant stroma and heterogeneous vascular permeability and density [3,4,5]. In these tumor tissues, the EPR effect is negligible, resulting in an insufficient intratumoral distribution of nanoparticles.

For effective chemotherapy against such intractable cancers, cells such as mesenchymal stem cells (MSCs), macrophages, and neutrophils have attracted increasing attention as promising carriers for active drug delivery systems targeting tumors. These cells possess an intrinsic ability to penetrate physiological barriers and accumulate in tumor tissue [6,7,8]. Among these cells, MSCs are commonly used in research on the development of tumor-targeted drug delivery systems because MSCs have several advantages for clinical use compared with other cells [9,10,11]. For example, MSCs can be collected from adult tissues, such as bone marrow, adipose tissue, and peripheral blood, and are easily cultured and expanded in vitro [12,13]. Moreover, MSCs show minimal immunogenicity, and therefore, allogeneic MSCs are suitable for clinical applications [12,13].

To prepare anti-cancer drug-loaded MSCs, anti-cancer drugs are frequently introduced into MSCs using nanoparticulate formulations [14,15,16], which avoids direct exposure of MSCs to anti-cancer drugs. However, since MSCs are non-phagocytic, their uptake efficiency of nanoparticles is presumed to be lower compared to phagocytic cells, such as macrophages and neutrophils [17,18]. Moreover, although anti-cancer drugs are encapsulated into nanoparticles, cell damage caused by the cellular uptake of the nanoparticles is inevitable. Therefore, to develop anti-cancer drug-loaded MSCs with minimal cytotoxicity, loading nanoparticles encapsulated in anti-cancer drugs onto the cell surface rather than inside the cells may be beneficial.

Several methods for loading nanoparticles onto the surface of MSCs have been reported [19,20,21]. In these reports, nanoparticles are bound to the cell surface using antigen–antibody or avidin–biotin interactions. Unlike these methods, we previously developed a technique for the surface modification of MSCs with liposomes via electrostatic interactions by using magnetic anionic liposome/atelocollagen (Mag-AL/ATCOL) complexes [22,23]. Our method enables efficient liposome loading onto the surface of MSCs within 30 min. Moreover, Mag-AL/ATCOL complexes bound to the surface of MSCs was stably retained for at least 24 h in the absence of a magnetic field, without significant detachment and cellular internalization. We also showed that the constructed liposome-loaded MSCs (Lip-MSCs) efficiently accumulate in B16/BL6 melanoma tumors with low vascular permeability while stably carrying liposomes [22].

In this study, we prepared doxorubicin (DOX)-encapsulated Mag-AL (DOX-Mag-AL)/ATCOL complexes and evaluated the DOX loading efficiency, cytotoxicity, and DOX-releasing properties when the complexes were loaded onto the surface of MSCs. The tumor-tropic capacity and anti-tumor efficacy of DOX-Mag-AL/ATCOL complex-loaded MSCs (DOX-Lip-MSCs) were also investigated.

## 2. Materials and Methods

### 2.1. Cell Culture

C57BL/6 mouse MSCs were obtained from Cyagen Biosciences Inc. (Santa Clara, CA, USA) and grown in Mouse MSC Growth Medium (Cyagen Biosciences Inc.). MSCs were used within five passages in all experiments. B16/BL6 murine melanoma cells were kindly gifted from the Cell Resource Center for Biomedical Research, Institute of Development, Aging and Cancer, Tohoku University (Sendai, Japan). B16/BL6 cells were cultivated in RPMI-1640 medium containing 10% heat-inactivated fetal bovine serum (FBS), penicillin G (100 U/mL), and streptomycin (100 µg/mL). All cells were maintained at 37 °C in a humidified 5% CO_2_ atmosphere.

### 2.2. Animals

Six-week-old male C57BL/6N mice were purchased from Japan SLC (Hamamatsu, Japan). All animal experiments were conducted according to the principles and procedures outlined in the Guide for the Care and Use of Laboratory Animals (National Institutes of Health, Bethesda, MD, USA). The study protocol was approved by the Kobe Pharmaceutical University Committee for Animal Care and Use (approval number: 2023-009). All efforts were made to minimize suffering.

### 2.3. Preparation of DOX-Mag-AL/ATCOL Complexes

Mag-AL composed of 1,2-distearoyl-*sn*-glycero-3-phospho-(1′-*rac*-glycerol) (NOF Inc., Tokyo, Japan), cholesterol (Nacalai Tesque Inc., Kyoto, Japan), iron oxide (II, III) magnetic nanoparticles (Sigma-Aldrich, St. Louis, MO, USA), and DiIC18 (3) (Wako Pure Chemical Industries, Ltd., Osaka, Japan) was prepared with different inner aqueous phases (pH 5.0–6.5 citrate buffer containing 0–250 mM (NH_4_)_2_SO_4_), according to our previous report [24]. The prepared Mag-AL was sonicated for 3 min or 30 sec using a probe-type sonicator to obtain small- or large-sized Mag-AL, respectively. Mag-AL/ATCOL complexes were formed by gently mixing 100 µg lipid/mL of Mag-AL with an equal volume of 40 µg/mL ATCOL (Koken Co., Ltd., Tokyo, Japan), followed by incubation for 20 min at 4 °C. The particle sizes and ζ-potentials of the prepared Mag-AL were measured using a Zetasizer Pro (Malvern Instruments, Worcestershire, UK).

DOX was loaded into Mag-AL using the pH-gradient and/or (NH_4_)_2_SO_4_ concentration gradient remote-loading method [25]. The encapsulated DOX ratio in Mag-AL was measured by HPLC.

### 2.4. Cellular Association of Mag-AL/ATCOL Complexes in MSCs

MSCs were seeded in 24-well culture plates at a density of 2.5 × 10^4^ cells/cm^2^ and cultured for 72 h. Small-sized Mag-AL or Mag-AL/ATCOL complexes (25 µg of lipid) were added to each well and incubated for 30 min at 37 °C under magnetic field using a magnetic plate (OZ Biosciences, San Diego, CA, USA). The cells were washed twice with phosphate-buffered saline (PBS) and lysed with lysis buffer (0.5% Triton X-100, 2 mM ethylenediaminetetraacetic acid, 0.1 M Tris, pH 7.8). The resultant lysates were centrifuged at 10,000× *g* for 10 min at 4 °C, and then, the amount of Mag-AL in the supernatant was quantified by measuring the fluorescence intensity using a Synergy HTX multimode microplate reader (Agilent Technologies Japan, Ltd., Tokyo, Japan).

### 2.5. Cytotoxicity and Association of DOX-Mag-AL/ATCOL Complexes with MSCs

MSCs cultured in 24-well culture plates were incubated with DOX solution or DOX-Mag-AL/ATCOL complexes at a DOX concentration of 0.5–20 µg/mL for 30 min at 37 °C on a magnetic plate. The cells were washed twice with PBS, and cell viability was measured using Cell Counting Reagent SF (Nacalai Tesque Inc.) and a Synergy HTX multimode microplate reader. Acetonitrile was added to the well to extract DOX from the cells. The DOX concentration in each sample was quantified by HPLC.

### 2.6. DOX Release from DOX-Lip-MSCs

To prepare DOX-Lip-MSCs, MSCs were cultured in a 100 mm culture dish and incubated with DOX-Mag-AL/ATCOL complexes at a DOX concentration of 10 µg/mL for 30 min at 37 °C on a magnetic plate. The cells were then washed twice with PBS, and fresh medium containing 50% FBS was added to the dish. The cell culture supernatant was collected at predetermined time points, and the DOX concentration was measured by HPLC.

### 2.7. In Vitro Migration of DOX-Lip-MSCs

Non-loaded MSCs or DOX-Lip-MSCs (1 × 10^5^ cells) were suspended in serum-free medium and added to the upper compartment of 12-well cell culture inserts (8 µm pore size; Corning Life Sciences, Corning, NY, USA). The lower compartment was filled with RPMI-1640 medium containing 0.5% FBS (normal medium) or conditioned RPMI-1640 medium containing 0.5% FBS from B16/BL6 cells. The cells in the cell culture inserts were incubated for 24 h at 37 °C and then stained with calcein AM and fixed in 4% paraformaldehyde. The cells attached to the upper surface of the membrane were removed with cotton swabs, and the lower surface of the membrane was observed using a BZ-X810 fluorescence microscope (KEYENCE Corporation, Tokyo, Japan).

For evaluating the cytotoxic effects of DOX-Lip-MSCs against B16/BL6 cells, B16/BL6 cells were seeded in the lower compartment of 12-well cell culture inserts at a density of 2 × 10^4^ cells/cm^2^ and cultured for 24 h. Then, non-loaded MSCs or DOX-Lip-MSCs (1 × 10^5^ cells) suspended in serum-free medium were added to the upper compartment, and the culture medium in the lower compartment was replaced with normal medium or conditioned medium from B16/BL6 cells. The cells in cell culture inserts were incubated for 24 h and 48 h at 37 °C, and cell viability of B16/BL6 cells was measured using Cell Counting Reagent SF and a Synergy HTX multimode microplate reader.

### 2.8. In Vivo Tissue Distribution of DOX-Lip-MSCs

DOX-Mag-AL was radiolabeled with indium-111 (^111^In) using the remote-loading method according to our previous report [22]. In brief, Mag-AL was prepared with inner aqueous phase of pH 6.0 citrate buffer containing 250 mM of (NH_4_)_2_SO_4_ and 10 mM of diethylenetriamine-*N*,*N*,*N*′,*N*″,*N*″-pentaacetic acid. The prepared Mag-AL was mixed with 51 mM of oxine and 1 mg/mL of DOX in ethanol containing ^111^In-Cl_3_, and incubated for 30 min at 60 °C. Then, the excess ^111^In-Cl_3_ and DOX were removed by gel filtration using the Sephadex G-50 gel column (Global Life Sciences Technologies Japan K.K., Tokyo, Japan). To prepare tumor-bearing mice, mice were subcutaneously injected with 1 × 10^6^ B16/BL6 cells. When the tumor volume reached 500 mm^3^, ^111^In-labeled DOX-Lip-MSCs (1.5 × 10^6^ cells) or the corresponding lipid amount of ^111^In-labeled DOX-Mag-AL were intravenously administered into tumor-bearing mice. At 24 h and 48 h after administration, the mice were sacrificed, and the heart, lung, kidney, spleen, liver, and tumor were harvested. The amount of DOX-Mag-AL in each organ was quantified by measuring the radioactivity using a PerkinElmer 2480 WIZARD2 automatic gamma counter (PerkinElmer Japan Co. Ltd., Kanagawa, Japan).

### 2.9. In Vivo Anti-Tumor Efficacy of DOX-Lip-MSCs

When the tumor volume of B16/BL6 tumor-bearing mice reached 100 mm^3^, saline, MSCs (1.5 × 10^6^ cells), DOX solution, DOX-Mag-AL, or DOX-Lip-MSCs (1.5 × 10^6^ cells) were intravenously injected into the mice at a dose of 0.5 mg DOX/kg three times (days 0, 3, and 6). The tumor volume and body weight were recorded every other day. The experiment was terminated on day 12 after the first treatment, when the tumor volume in saline-treated mice reached 10% of the body weight (2500 mm^3^).

### 2.10. HPLC Measurement of DOX

DOX was extracted from each sample according to our previous report [22]. Briefly, 0.1 mL of each sample was mixed with 1.0 mL acetonitrile. The mixture was centrifuged at 10,000× *g* for 10 min at 4 °C, and the supernatant was collected and evaporated. The residue was re-dissolved in the HPLC mobile phase (1:15 M KH_2_PO_4_:CH_3_CN = 75:25 (*v*/*v*, pH 4.16, adjusted with H_3_PO_4_)). The concentration of DOX was measured using an HPLC system comprising an LC-20AD pump, SIL-20A autosampler, RF-20A fluorescence detector, CMB-20A system controller (Shimadzu, Kyoto, Japan), and ODS column (5C_18_, 150 mm × 4.6 mm i.d., Nacalai Tesque Inc.). The mobile phase was delivered at 1.0 mL/min. DOX was detected by measuring the fluorescence intensity (excitation: 470 nm, emission: 550 nm).

### 2.11. Statistical Analysis

The results are presented as the mean + or ± standard deviation (SD) of three to six experiments. Analysis of variance (ANOVA) was used to test the statistical significance of differences between groups. Two-group comparisons were performed using Student’s *t*-test. Multiple comparisons between all groups were performed using the Tukey–Kramer test.

## 3. Results

### 3.1. Optimization of the Composition and Particle Size of Mag-AL for Efficient and Stable Remote Loading of DOX

#### 3.1.1. Effect of the Inner Aqueous Phase of Mag-AL on the Cellular Association and DOX Retention of Mag-AL/ATCOL Complexes

**Figure 1:**
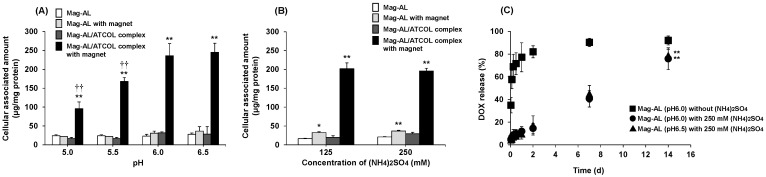
Cellular association and DOX-releasing properties of small-sized Mag-AL/ATCOL complexes with different inner aqueous phase compositions. Mag-AL was prepared by hydration with pH 5.0–6.5 citrate buffer containing 250 mM (NH_4_)_2_SO_4_ (**A**) or pH 6.0 citrate buffer containing 125 or 250 mM (NH_4_)_2_SO_4_ (**B**). Mag-AL/ATCOL complexes were added to each well and incubated for 30 min at 37 °C in the presence or absence of a magnetic field. Each value represents the mean + SD (*n* = 4). * *p* < 0.05; ** *p* < 0.01, compared with the value in the absence of a magnetic field. ^††^ *p* < 0.01, compared with the corresponding group at pH 6.5. (**C**) Small-sized DOX-Mag-AL with an inner aqueous phase at pH 6.0 or 6.5 containing 0 or 250 mM (NH_4_)_2_SO_4_ was mixed with an equal volume of FBS in a dialysis bag and incubated for 14 days at 37 °C with shaking. The amount of DOX released was measured by HPLC. Each value represents the mean ± SD (*n* = 4). ** *p* < 0.01, compared with Mag-AL (pH 6.0) without (NH_4_)_2_SO_4_.

The (NH_4_)_2_SO_4_ concentration and pH in the intraliposomal aqueous phase are critical factors for efficient remote loading of DOX [26,27]. However, it is unclear whether these factors affect the magnetic responsivity of Mag-AL. Therefore, we prepared several small-sized Mag-AL with different inner aqueous phase compositions and evaluated their cellular association with MSCs. The particle sizes and ζ-potentials were approximately 110 nm and −35 mV, respectively (Table 1). Regardless of the pH in the intraliposomal aqueous phase, the amount of cell-associated Mag-AL/ATCOL complexes on MSCs was remarkably increased by the presence of a magnetic field (Figure 1A). However, the association was significantly lower in the complexes with an intraliposomal pH of 5.0 or 5.5 compared with those with an intraliposomal pH of 6.0 or 6.5. The concentration of (NH_4_)_2_SO_4_ in the aqueous phase of Mag-AL did not affect the cellular association of the complexes (Figure 1B).

Next, DOX was loaded into small-sized Mag-AL with different internal aqueous phase compositions, and the DOX-releasing properties from Mag-AL were assessed. The encapsulation efficiency was over 95% in all the small-sized Mag-AL (Table 1). As shown in Figure 1C, DOX was rapidly released from Mag-AL without (NH_4_)_2_SO_4_ in the presence of FBS, whereas Mag-AL containing (NH_4_)_2_SO_4_ exhibited a sustained DOX release, which is likely caused by the gelation of DOX with sulfate in the aqueous phase of Mag-AL [27]. There was no significant difference in the DOX release profiles between Mag-AL with intraliposomal pH values of 6.0 and 6.5. The DOX release from Mag-AL was approximately 15% at day 2, increased to 40% at day 7, and reached approximately 80% at day 14.

#### 3.1.2. Effect of the Particle Size of Mag-AL on DOX Retention Properties

**Figure 2:**
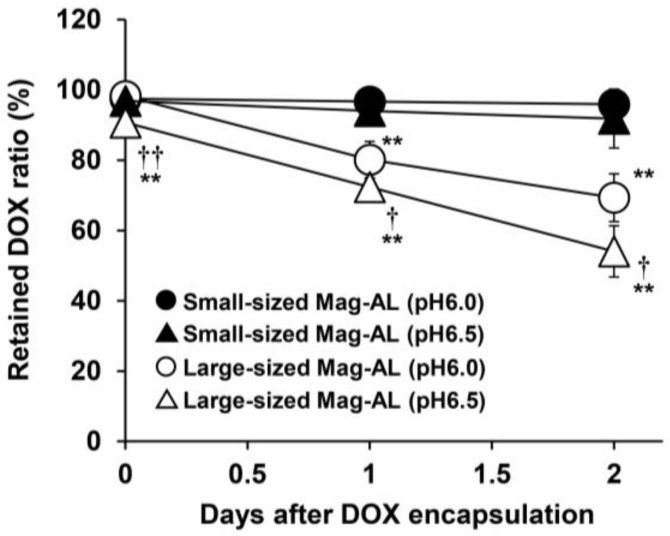
DOX retention efficiency of Mag-AL. Small- and large-sized Mag-AL with an inner aqueous phase of pH 6.0 or 6.5 were stored for 2 days at 4 °C. The amount of retained DOX in each Mag-AL was measured by HPLC at days 0, 1, and 2. Each value represents the mean ± SD (*n* = 4). ** *p* < 0.01, compared with the corresponding group of small-sized Mag-AL. ^†^ *p* < 0.05; ^††^ *p* < 0.01, compared with the corresponding group at pH 6.0.

We next investigated the effect of the particle size of Mag-AL on the DOX retention efficiency. When small-sized DOX-Mag-AL complexes were stored for 2 days under storage conditions (4 °C, FBS-free), DOX was stably retained inside Mag-AL (Figure 2). We also found that small-sized Mag-AL could stably retain DOX for at least 2 weeks. In contrast, large-sized DOX-Mag-AL (particle size: approximately 600 nm, Table 1) showed much lower stability, and the retained DOX ratio was less than 80% after 2 days of storage.

### 3.2. DOX Loading Amount, Cell Viability, and DOX-Releasing Properties of DOX-Lip-MSCs

**Figure 3:**
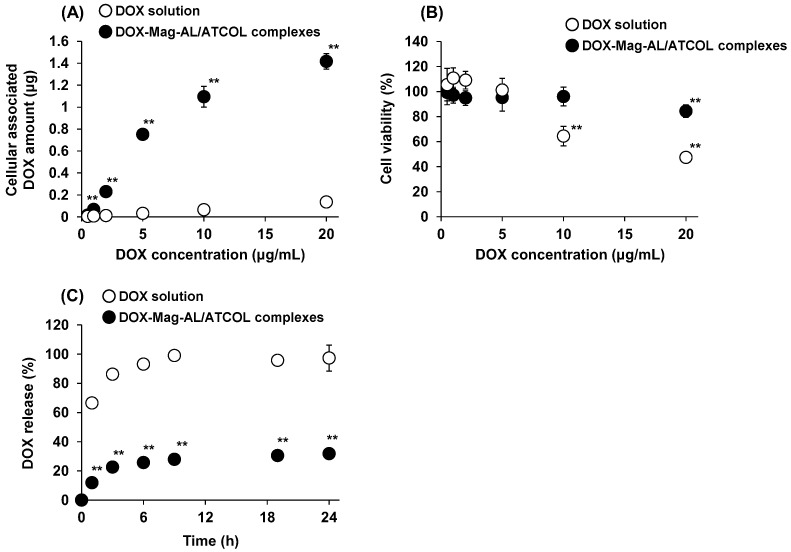
DOX loading amount, cell viability, and DOX-releasing properties of DOX-Lip-MSCs. MSCs were incubated with 0.5–20 µg DOX/mL of DOX solution or DOX-Mag-AL/ATCOL complexes for 30 min in the presence of a magnetic field. (**A**) The amount of DOX loaded on MSCs was measured. Each value represents the mean ± SD (*n* = 4). ** *p* < 0.01, compared with the DOX solution. (**B**) Cell viability was measured. Each value represents the mean ± SD (*n* = 4). ** *p* < 0.01, compared with untreated MSCs. (**C**) MSCs were incubated with DOX solution (5 µg DOX/mL) or DOX-Mag-AL/ATCOL complexes (10 µg DOX/mL) for 30 min in the presence of a magnetic field. The amount of DOX released from the cells into the medium was measured. Each value represents the mean ± SD (*n* = 4). ** *p* < 0.01, compared with the DOX solution.

A quantitative analysis of the amount of DOX loaded on MSCs using DOX-Mag-AL/ATCOL complexes was performed. The amount of DOX loaded on MSCs was significantly larger when the surface of MSCs was modified with DOX-Mag-AL/ATCOL complexes than when MSCs were incubated with DOX solution (Figure 3A). A DOX concentration-dependent increase in the cellular load of DOX was also observed when MSCs were modified with DOX-Mag-AL/ATCOL complexes. In addition, the cytotoxicity associated with the DOX loading was assessed. As shown in Figure 3B, a significant reduction in cell viability was observed following incubation of MSCs in DOX solution. In contrast, the viability of MSCs modified with Dox-Mag-AL/ATCOL complexes was maintained at over 95% up to 10 µg/mL DOX. Then, the phenotypic changes of MSCs by the DOX loading were also evaluated. We confirmed that DOX-Lip-MSCs expressed comparable levels of the MSC surface markers, CD29 and CD44, to those of non-loaled MSCs (Supplementary Figure S1A,B). Moreover, the expression of CD31 was not detected in both non-loaded MSCs and DOX-Lip-MSCs. In addition, the production levels of inflammatory cytokines (TNF-α and IL-6) and anti-inflammatory cytokine (IL-10) were not significantly different from those of non-loaded MSCs (Supplementary Figure S1C–E).

Next, we evaluated the release of DOX from DOX-Lip-MSCs. DOX was rapidly released from DOX-loaded MSCs prepared by incubating MSCs with DOX solution, and approximately 100% of DOX was released within 9 h (Figure 3C). In contrast, a sustained release of DOX was observed with DOX-Lip-MSCs.

### 3.3. In Vitro and In Vivo Tumor-Tropic Ability of DOX-Lip-MSCs

**Figure 4:**
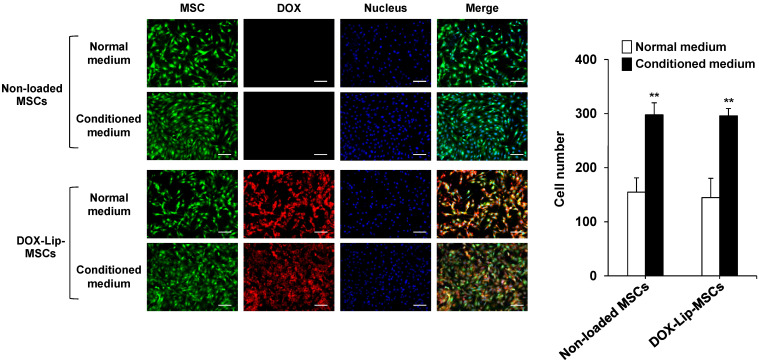
In vitro migration of DOX-Lip-MSCs. (**A**) Representative photographs of non-loaded MSCs or DOX-Lip-MSCs that migrated through the membrane pores toward the normal medium or conditioned medium of B16/BL6 cells. Scale bar: 100 µm. (**B**) Histogram showing the number of migrated cells in both non-loaded MSCs and DOX-Lip-MSCs in normal and conditioned medium. Each value represents the mean + SD (*n* = 3). ** *p* < 0.01, compared with cells in normal medium.

The in vitro tumor-tropic potential of DOX-Lip-MSCs was investigated. The number of DOX-Lip-MSCs that migrated toward the conditioned medium of B16/BL6 cells was significantly higher than the number that migrated toward the normal unconditioned medium (Figure 4). There was no significant difference in the migratory behavior between non-loaded MSCs and DOX-Lip-MSCs.

**Figure 5:**
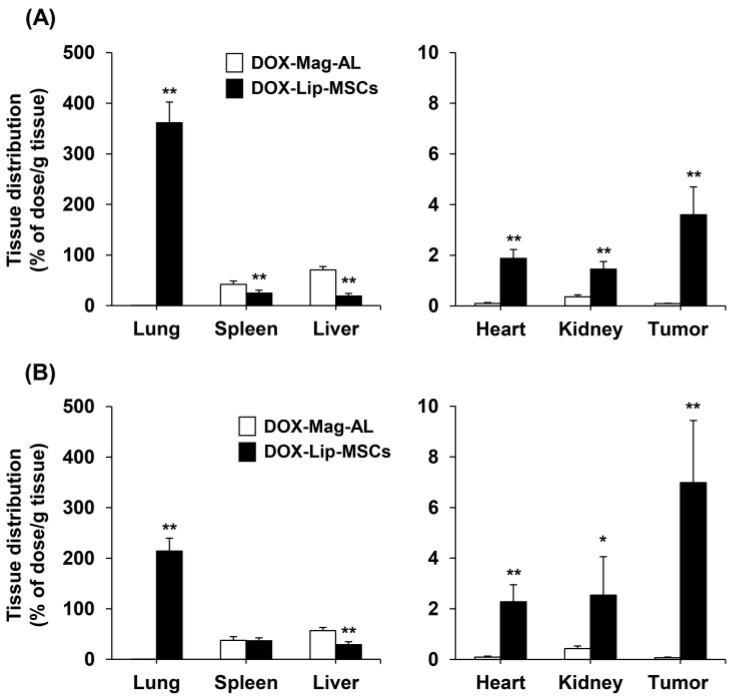
In vivo tissue distribution of DOX-Lip-MSCs in B16/BL6 tumor-bearing mice. ^111^In-labeled DOX-Lip-MSCs (1.5 × 10^6^ cells) or the corresponding lipid amount of ^111^In-labeled DOX-Mag-AL were intravenously injected into B16/BL6 tumor-bearing mice. The accumulated amount of Mag-AL in the heart, lung, kidney, spleen, liver and tumor was quantified at 24 h (**A**) and 48 h (**B**) after injection. Each value represents the mean + SD (*n* = 4). * *p* < 0.05; ** *p* < 0.01, compared with DOX-Mag-AL.

We also assessed the in vivo tissue distribution of DOX-Lip-MSCs after intravenous administration into B16/BL6 tumor-bearing mice. As shown in Figure 5, DOX-Mag-AL predominantly accumulated in the liver and spleen at 24 h after intravenous injection. In contrast, DOX-Lip-MSCs were preferentially distributed in the lungs at 24 h after injection, but then moved and accumulated in the liver and spleen. In the tumors, the accumulated amount of DOX-Lip-MSCs was approximately 40-fold greater than that of DOX-Mag-AL at 24 h after administration, and 100-fold greater at 48 h after administration.

### 3.4. In Vitro and in Vivo Anti-Tumor Efficacy of DOX-Lip-MSCs

**Figure 6:**
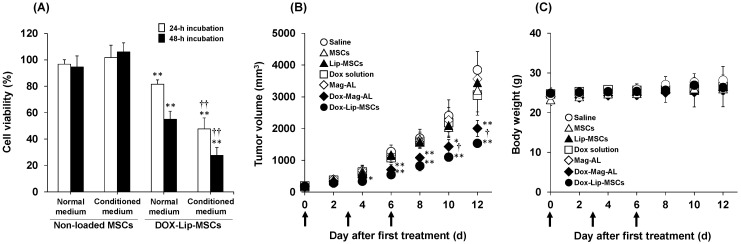
In vitro and in vivo anti-tumor efficacy of DOX-Lip-MSCs in B16/BL6 tumor-bearing mice. (**A**) Histogram showing the viability of B16/BL6 cells co-cultured with either non-loaded MSCs or DOX-Lip-MSCs in normal or conditioned medium from B16/BL6 cells. B16/BL6 cells were seeded in the lower compartment of cell culture inserts, while non-loaded MSCs or DOX-Lip-MSCs (1 × 10^5^ cells), suspended in serum-free medium, were added to the upper compartment. The co-culture was incubated at 37 °C for 24 or 48 h, after which the viability of B16/BL6 cells was assessed. Each value represents the mean ± SD (*n* = 6). ** *p* < 0.01, compared with non-loaded MSCs. ^††^ *p* < 0.01, compared with normal medium. (**B**) Tumor volume and (**C**) body weight of B16/BL6 tumor-bearing mice intravenously treated with saline, MSCs, DOX solution, DOX-Mag-AL, or DOX-Lip-MSCs. Treatments were administered on days 0, 3, and 6, with DOX dosed at 0.5 mg/kg. Tumor volume and body weight were measured every other day. Each value represents the mean ± SD (*n* = 6). * *p* < 0.05; ** *p* < 0.01, compared with saline. ^†^ *p* < 0.05, compared with DOX-Mag-AL.

We next assessed the in vitro cytotoxic effects of DOX-Lip-MSCs against B16/BL6 cells by co-culturing these cells using cell culture inserts. As shown in Figure 6A, DOX-Lip-MSCs showed cytotoxicity against B16/BL6 cells, and it was significantly increased when B16/BL6 cells were cultured in conditioned medium of B16/BL6 cells. Then, the in vivo anti-tumor efficacy of DOX-Lip-MSCs was also investigated in B16/BL6 tumor-bearing mice. DOX solution exhibited no anti-tumor efficacy, whereas DOX-Mag-AL effectively inhibited tumor growth (Figure 6B). The most significant anti-tumor efficacy was observed with DOX-Lip-MSCs (Figure 6B), and DOX-Lip-MSCs did not cause significant body weight loss (Figure 6C).

## 4. Discussion

The cytotoxic effects of DOX against MSCs would cause a loss of their tumor-tropic ability and therefore pose a major obstacle to the use of MSCs as carriers for active drug delivery systems. In this study, to overcome this obstacle, DOX was loaded on the surface of MSCs, rather than inside the cells, by using Mag-AL/ATCOL complexes.

We first optimized the composition of the inner aqueous phase of Mag-AL for efficient and stable DOX loading. The pH gradient between the inside and outside of Mag-AL directly affects the remote-loading efficiency, and the intraliposomal pH is commonly set at around 5.5 for the remote-loading of DOX. However, our results showed that the magnetic responsivity of Mag-AL was significantly decreased when the intraliposomal pH was less than or equal to 5.5 (Figure 1A). The brownish solution of iron oxide magnetic nanoparticles that was used as the intraliposomal aqueous phase became transparent at pH 5.5 and below. Moreover, we also observed that the particle size of magnetic nanoparticles at pH6.0 was 17.8 ± 5.0 nm, whereas the particles could not be detected at pH5.5. These results suggest that the magnetic nanoparticles collapsed under these acidic conditions. Considering these results, the pH in the inner aqueous phase of Mag-AL was set to 6.0. Although this intraliposomal pH was relatively higher, the encapsulation efficiency of DOX in Mag-AL containing 250 mM (NH_4_)_2_SO_4_ was over 95% and its in vitro DOX release profile (Figure 1C) was similar to that of anionic liposomes with the inner aqueous phase composed of 250 mM (NH_4_)_2_SO_4_ at pH 4.5 that we previously reported [25]. These results indicate that the remote-loading efficiency and release properties of DOX in Mag-AL are determined by the intraliposomal concentration of (NH_4_)_2_SO_4_, rather than the pH.

We also demonstrated that small-sized Mag-AL had a much higher DOX retention capacity compared with large Mag-AL (Figure 2). Our previous report demonstrated that small-sized Mag-AL/ATCOL complexes tend to be internalized into MSCs. However, its internalized ratio was not markedly different from that of the large-sized complexes. Moreover, the number of cell-associated small-sized complexes in MSCs was higher than that of the large-sized complexes [22]. Based on these findings, we consider that small-sized Mag-AL/ATCOL complexes are suitable for efficient DOX loading on the surface of MSCs.

Small-sized DOX-Mag-AL/ATCOL complexes can achieve a significantly higher DOX loading on MSCs with minimal reduction in cell viability compared with DOX solution (Figure 3A,B). Since it has been reported that even a small amount of DOX induces severe functional abnormalities, such as mitochondrial dysfunction and senescence, in MSCs [28,29,30], a more detailed investigation regarding the cytotoxicity in DOX-Lip-MSCs is needed. Nevertheless, these results demonstrate that the loading of DOX onto the surface of MSCs using Mag-AL/ATCOL complexes could dramatically reduce the cytotoxic effects of DOX against MSCs. The estimated DOX content on DOX-Lip-MSCs was 6.2 pg/cell. Zhang et al. reported that the DOX content in DOX-polymer conjugate-internalized MSCs was 5.8 pg/cell [31]. Sadhukha et al. showed that the paclitaxel content in MSCs that had taken up paclitaxel-loaded PLGA nanoparticles was 4.7 pg/cell [32]. Thus, the drug loading efficiency on MSCs by cell surface modification with Mag-AL/ATCOL complexes is comparable to that in MSCs loaded by the cellular internalization of nanoparticles.

The constructed DOX-Lip-MSCs showed much slower DOX release than MSCs loaded with DOX by incubating the cells in DOX solution (Figure 3C). The rapid DOX release from DOX-loaded MSCs prepared by incubation with DOX solution is likely because of the active efflux of DOX mediated by P-glycoprotein (P-gp), which is expressed by MSCs [32,33]. In contrast, the release rate of DOX from DOX-Lip-MSCs (Figure 3C) was faster than that from DOX-Mag-AL (Figure 1C). This may be because a portion of DOX-Mag-AL/ATCOL complexes on the surface of MSCs was taken up by the cells. The internalized DOX-Mag-AL in MSCs would be digested, and the encapsulated DOX would be rapidly released extracellularly, mediated by P-gp.

Tumor-tropic ability is a major requirement for using MSCs as drug carriers. Our results demonstrate that DOX-Lip-MSCs preferentially migrated toward tumor cell-conditioned medium in vitro and accumulated in the tumor in vivo (Figure 4 and Figure 5). The in vivo tumor accumulation of DOX-Lip-MSCs at 48 h after intravenous injection (6.2% ± 1.9% of dose/g tumor) was similar to that of empty Lip-MSCs (7.1% ± 1.8% of dose/g tumor) that we previously reported [19]. These observations indicate that DOX does not affect the tumor-tropic capacity of Lip-MSCs. Owing to this favorable kinetic property of DOX-Lip-MSCs, a significant inhibitory effect of DOX on tumor growth was obtained (Figure 6B). However, in contrast to expectations, the anti-tumor efficacy of DOX-Lip-MSCs was not dramatically greater than that of DOX-Mag-AL, despite the superior tumor accumulation of DOX-Lip-MSCs compared with DOX-Mag-AL. It has been reported that naked liposomes are distributed in the tumor tissue within 6 h after intravenous administration into tumor-bearing mice and then eliminated from the tumor tissue by 24 h after administration [34,35]. Based on this information, we assume that the accumulated DOX-Mag-AL in the tumor immediately after administration exhibits anti-tumor efficacy, although its retention time in tumor tissue is relatively brief.

A feasible means to enhance the anti-tumor efficacy of DOX-Lip-MSCs would be to circumvent the lung entrapment. Lung entrapment of intravenously injected MSCs is caused by microembolization with MSCs in pulmonary capillaries [36,37], and approximately 50% of the injected DOX-Lip-MSCs were entrapped in the lungs at 24 h after injection (Figure 5A). Takayama et al. demonstrated that surface modification of MSCs with polyethylene glycol significantly suppresses the lung entrapment of intravenously injected MSCs in mice [38]. Moreover, Schrepfer et al. showed that embolization with MSCs in the murine lung can be diminished by vasodilation in response to pretreatment with sodium nitroprusside [39]. These approaches may enhance the tumor accumulation and anti-tumor efficacy of DOX-Lip-MSCs.

## 5. Conclusions

DOX was loaded onto MSCs without significant cytotoxicity by cell surface modification with Mag-AL/ATCOL complexes. Moreover, MSCs significantly enhanced the tumor accumulation of DOX-Lip at 24 and 48 h after intravenous injection. Onf the other hand, although the anti-tumor efficacy of DOX-Lip-MSCs was not markedly higher than that of DOX-Mag-AL, preventing the lung entrapment of DOX-Lip-MSCs would likely lead to an improvement in its therapeutic efficacy. Since liposomes are capable of loading not only low-molecular-weight drugs but also peptides, proteins, and nucleic acids, Lip-MSCs have the potential to serve as a versatile carrier for drug delivery systems targeting tumors.

## Figures and Tables

**Table 1:** Physicochemical properties and DOX encapsulation efficiency of Mag-AL.

	pH	(NH_4_)_2_SO_4_ Concentration (mM)	Particle Size (nm)	ζ-Potential (mV)	Polydispersity Index (PDI)	DOX Encapsulation
Small-sized	5.0	250	111.4 ± 6.4	−37.2 ± 1.7	0.14 ± 0.05	NE
	5.5	250	107.6 ± 2.3	−36.4 ± 2.3	0.17 ± 0.03	NE
	6.0	125	117.0 ± 5.5	−34.4 ± 4.1	0.11 ± 0.02	96.2 ± 2.0
	6.0	250	111.6 ± 9.3	−38.3 ± 1.7	0.15 ± 0.04	97.5 ± 1.3
	6.5	250	106.0 ± 4.2	−33.1 ± 2.5	0.19 ± 0.04	96.9 ± 1.3
Large-sized	6.0	250	634.4 ± 15.0	−36.6 ± 3.1	0.32 ± 0.03	98.3 ± 0.8
	6.5	250	618.0 ± 17.2	−36.8 ± 0.9	0.31 ± 0.06	90.7 ± 3.7

Each value represents the mean ± SD (*n* = 3). NE: Not evaluated.

## Data Availability

All relevant data are included in the manuscript.

## Supplementary Materials

The following supporting information can be downloaded at: https://www.mdpi.com/article/10.3390/pharmaceutics17080947/s1, Figure S1: The expression of surface markers and cytokines in DOX-Lip-MSCs.
